# Investigation of Axial Tensile Fracture Performance of Recycled Brick Coarse Aggregate Concrete Using a Cohesion Model

**DOI:** 10.3390/ma17153630

**Published:** 2024-07-23

**Authors:** Yu Zeng, Qionglin Li, Zhenchao Yang, Qilong Zhao

**Affiliations:** 1 School of Civil Engineer and Architecture, Southwest University of Science and Technology, Mianyang 621010, China; yzczc992@163.com (Z.Y.); cirenwd1999@163.com (Q.Z.); 2 Shock and Vibration of Engineering Materials and Structures Key Laboratory of Sichuan Province, Mianyang 621010, China; 3 School of Civil Engineering, Southwest Jiaotong University, Chengdu 610031, China

**Keywords:** recycled brick coarse aggregate, tensile fracture performance, cohesive zone model, fracture process, pore structure

## Abstract

Currently, microscopic research on the tensile fracture properties of recycled brick coarse aggregate concrete has mainly adopted microscopy techniques, which can clearly observe the actual damage situations of each phase material but are unable to individually analyze the effect of a specific material factor on the tensile properties of recycled concrete. This brings much uncertainty to the practical application of recycled concrete. Therefore, this study proposes a cohesive zone model (CZM) for simulating the tensile fracture of recycled brick coarse aggregate (RBCA) concrete. To this end, the study explores the effects of various critical factors on the fracture mode and bearing capacity of recycled brick aggregate concrete, including the replacement rate of recycled brick coarse aggregate, pore structure, interfacial transition zone (ITZ) strength, mortar strength, and volume fraction of brick aggregate. The results indicate that, when the minor to major axis ratio of elliptical pores is 0.5 ≤ K < 1, the following order of influence can be observed: random convex polygonal pores, circular pores, and elliptical pores. Moreover, excessively strengthening the ITZ and mortar does not significantly enhance the tensile performance of RBCA concrete. The distribution location of aggregate has a significant impact on the crack shape of recycled concrete, as does the pore structure, due to their randomness. Therefore, this article also discusses these. These findings contribute to a comprehensive understanding of the tensile properties of recycled brick coarse aggregate and provide insights into optimizing its behavior.

## 1. Introduction

With the accelerated urbanization in China, the consumption of concrete has been steadily increasing year by year. According to statistical data, global consumption of concrete reached nearly 17.5 billion tons in 2018 [1], while in 2021, the consumption of commercial concrete in China alone rose to 3.06 billion cubic meters [2]. This surge in demand has led to a dual challenge: the depletion of natural aggregates (NAs) due to large-scale mining, and the accumulation of construction waste resulting from the demolition of old buildings, primarily composed of concrete and clay bricks. Currently, the predominant disposal methods for this waste involve dumping and landfilling, resulting in significant environmental repercussions [3,4,5]. To mitigate these issues, researchers have increasingly explored the use of various recycled aggregates (RAs). Over recent decades, a considerable amount of research has focused on substituting NAs with RAs in the production of recycled concrete (RC), leading to the establishment of standards such as GB/T 25177–2010 [6] “Recycled Coarse Aggregate for Concrete” and GB/T 25176–2010 [7] “Recycled Fine Aggregate for Concrete and Mortar”. Scholars generally agree that, when the replacement rate (RR) of waste concrete is below 30%, its impact on the mechanical properties of RC is minimal [8,9,10,11]. In practical engineering applications, RBCA concrete is primarily utilized for road bases. For road bases with moderate traffic volume, a mixture containing 15% crushed brick concrete is recommended; however, the use of crushed brick concrete for road bases is not advised for roads with high traffic volumes [12]. Research conducted by Poon et al. [13] has demonstrated the effective application of recycled coarse aggregates and crushed bricks in unbound road bases.

Given the significant influence of concrete’s tensile fracture performance on the performance of reinforced components, researchers have conducted numerous tensile tests on RBCA concrete. Liu et al. [14] found that, when the total replacement amount of recycled concrete coarse aggregate and RBCA did not exceed 30%, the inclusion of RBCA had no significant effect on the splitting tensile strength and flexural strength of RC. Similarly, Yang [15] conducted experiments with concrete made from recycled aggregates (RAs) and clay bricks, revealing that the content of broken clay bricks significantly impacts compressive strength and cylindrical splitting strength, while exerting a lesser influence on flexural strength. Moreover, increasing the content of broken clay bricks enhances concrete permeability. Additionally, Xu et al. [16] discovered that both the compressive strength and splitting tensile strength of RBCA concrete are lower than those of ordinary concrete. Collectively, these studies indicate that RBCA has a considerable impact on the tensile strength of concrete.

Furthermore, research on the microstructure of RC is burgeoning, aided by advancements in microscopic technology such as scanning electron microscopy (SEM), energy dispersive spectrometry (EDS), X-ray diffraction (XRD), mercury intrusion porosimetry (MIP), and backscattered electron (BSE) imaging [17,18,19,20]. These methods enable the analysis of different phase materials in RC. However, they cannot independently analyze the impact of a single material factor on the mechanical performance of RC, thus necessitating the use of finite element models (FEM).

In numerical modeling, RBCA concrete is viewed as a multiphase heterogeneous composite material composed of new mortar, old mortar, natural aggregates, brick aggregates, and the transition zones between the new and old interfaces. However, at a macroscopic level, elucidating the impact of the properties of different phase materials on the mechanical performance of RC presents challenges. Consequently, mesoscopic simulation has garnered significant attention, as it accurately explores the effects of different phase materials on the mechanical performance of RC under loading. For instance, Yu [21] developed a model of RC with circular aggregates, demonstrating that, as the replacement rate (RR) of recycled aggregates (RAs) increases, the elastic modulus of concrete decreases. Additionally, tensile strength is primarily influenced by the relative strength of the new and old mortars. Moreover, Wang [22] and Jin [23] employed Monte Carlo simulation for the tensile testing of RC, simulating crack propagation. Furthermore, other scholars have utilized methods such as peridynamics [24] and cohesive zone models [25,26,27,28] to simulate the expansion of internal cracks in concrete.

Scholars have studied the microstructure of three-dimensional concrete mesoscopic models [29,30], but the aggregate shapes used in these models are typically circular, which not only deviates from the actual shape but also incurs relatively high computational costs. Therefore, this study proposes a novel approach by developing a two-dimensional convex polygon random aggregate model. The objective is to systematically examine the influence of various factors on the tensile strength and fracture behavior of RC, incorporating Cohesive Interface Elements (CIEs) via a custom-written program. To ensure model accuracy, experimental methods adopted by other scholars will be utilized for model calibration.

This study aims to address several key objectives:

(i) Answer the question of how the replacement rate of RBCA impacts the tensile strength of concrete
(ii) Analyze the influence of pore structure, including porosity, pore size, and pore shape, on the tensile performance of RC.
(iii) Investigate whether the interfacial transition zone (ITZ) strength, the mortar strength, and the volume fraction of brick aggregate in concrete with full RBCA replacement affect the tensile strength of concrete.

Through comparative analysis of fracture patterns observed in the finite element model and collection of valuable data, this research endeavor aims to advance the design of brick aggregate concrete materials.

## 2. Establishment of the Cohesion Zone Model

### 2.1. Microscale Multiphase Models

**Figure 1:**
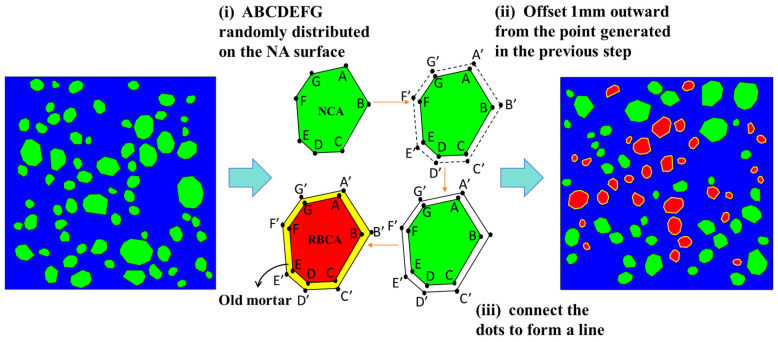
Natural coarse aggregate (NCA) and recycled brick coarse aggregate were generated.

From a microscopic perspective, ordinary concrete is a three-phase composite material composed of mortar, coarse aggregate, and ITZ, while RBCA concrete is a seven-phase composite material composed of NA, RBCA, old mortar, new mortar, new mortar–old mortar ITZ, NA-new mortar ITZ and RBCA–old mortar ITZ. This section establishes a mesoscopic model of natural aggregate concrete (NAC) and RBCA concrete with zero thickness ITZ and a geometric size of 150 mm × 150 mm. The shape of the aggregates is set to convex polygons. As the grading of aggregates affects the compactness of concrete, the grading curve with the maximum density, proposed by American scientist W.B Fuller in the early 20th century, is adopted [31]. This curve arranges the particles in a regular pattern and matches them evenly, making the prepared concrete the most compact. The expression is shown as Equation (1).

(1) $$P(D)=100\sqrt[{n}]{\frac{D_{0}}{D_{max}}}$$

where *P*(*D*) is the mass percentage of aggregates passing through sieve hole *D*_0_, *D*_0_ is the diameter of the sieve hole, *D_max_* is the maximum particle size of the aggregate, and *n* is the exponent of the equation (ranging from 0.45 to 0.70), which, in this paper, is chosen to be 0.5. The Fuller grading curve is applicable to three-dimensional space models, but, in this paper, a two-dimensional aggregate model is used. Therefore, the two-dimensional theoretical equation derived by Walraven and based on the Fuller equation is employed [32,33], and its expression is shown as Equation (2):

(2) $$P_{C}(D<D_{0})=P_{k}\left\{1.065{\left(\frac{D_{0}}{D_{max}}\right)}^{0.5}-0.053{\left(\frac{D_{0}}{D_{max}}\right)}^{4}-0.012{\left(\frac{D_{0}}{D_{max}}\right)}^{6}-0.0045{\left(\frac{D_{0}}{D_{max}}\right)}^{8}-0.0025{\left(\frac{D_{0}}{D_{max}}\right)}^{10}\right\}$$

where $P_{C}$(*D* < *D*_0_) is the probability that the aggregate particle size *D* within the cross-section is smaller than the sieve hole diameter *D*_0_ and $P_{k}$ represents the percentage of the aggregate e volume occupying the cross-sectional volume, with the rest being the same as in Equation (1). Based on Equations (1) and (2), the particle size ranges of RBCA are set to be 5 mm~10 mm, 10 mm~15 mm, and 15 mm~20 mm. The volume of aggregates typically accounts for 60%~75% of the total volume [34], of which coarse aggregates generally make up 40%~50% [35]. According to Equation (3), one can calculate the theoretical area of aggregates to be placed for each particle size range. Table 1 shows the aggregate particle size distribution rate for coarse aggregate volume fraction of 40%, generated using MATLAB-R2021b software.

(3) $$A_{k}=\left\{P_{k}(D<D_{k})-P_{k-1}(D<D_{k-1})\right\}\;\times \;A_{0}$$

where $A_{k}$ represents the theoretical area of aggregate placement for particle sizes within the range ($D_{k-1}$,$D_{k}$), $P_{k}(D<D_{k}$) represents the probability that the aggregate particle size D within the cross-section is smaller than the sieve range $D_{k}$, and $A_{0}$ represents the concrete cross-sectional area. The dimensions of the specimen cross-section are set to 150 mm × 150 mm, and a two-dimensional mesoscale model of concrete with a zero-thickness ITZ and a coarse aggregate volume fraction of 50% is shown in Figure 1.

### 2.2. Zero-Thickness Cohesion Interface Elements

**Figure 2:**
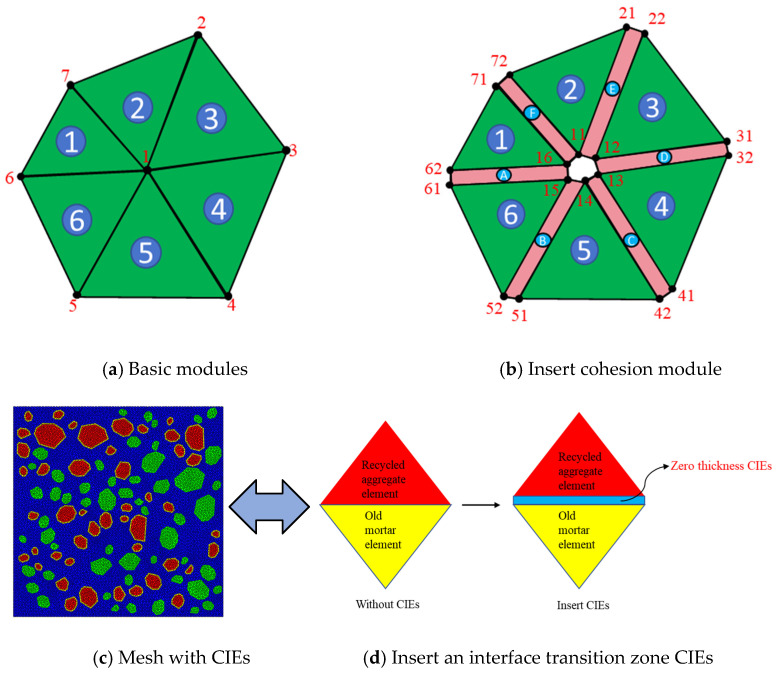
Schematic diagram of cohesion unit insertion.

**Figure 3:**
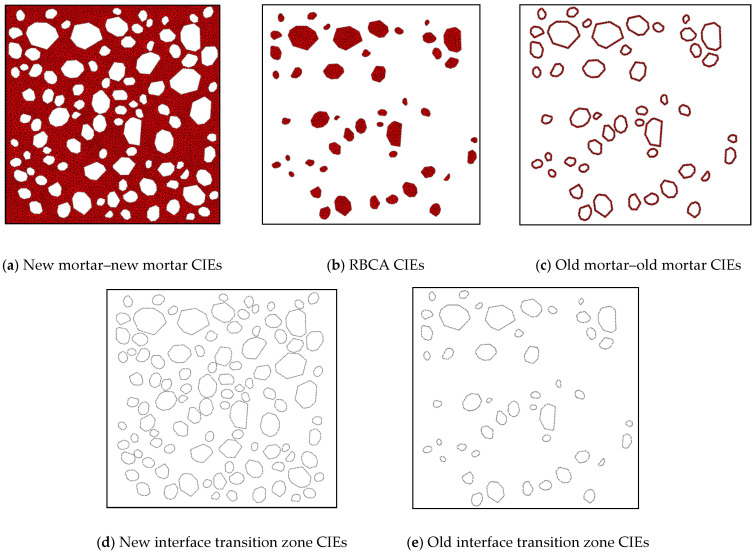
CIEs for each phase.

To accurately simulate the propagation of cracks in concrete under load, this study uses the ABAQUS CAE2022 software to establish a CZM. Inserting zero-thickness CIEs in potential crack regions and simulating the occurrence of cracks by removing the cohesive elements. This CZM is composed of seven phases: NCA, RBCA, old mortar, new mortar, zero-thickness RBCA–old mortar CIEs, zero-thickness new mortar–old mortar CIEs, and zero-thickness new mortar–natural aggregate CIEs. Before inserting the CIEs, it is first necessary to perform finite element mesh division on the mesoscopic model of concrete. The solid elements being triangular and the global seed size set to 2 mm. A Python (PyCharm Community Edition) script is used to locate each solid element, and zero-thickness CIEs are then defined by writing an inp file. The direction of element separation is set to y, that is, counter clockwise. Figure 2a shows a part of the basic elements, totaling six, with element ① represented as (1-2-7). If zero-thickness CIEs are to be inserted between element ① and ②, the CIEs cannot be represented as (1-7-7-1); instead, new nodes must be added, splitting node 1 into 11 and 16, and node 7 into 71 and 72, thus representing the zero-thickness CIEs as (11-72-71-16). The method of inserting zero-thickness CIEs between the other elements follows the same pattern. Figure 2b shows the zero-thickness CIEs after insertion, where basic elements ①–⑥ are split into CIEs A–F. Figure 2d exemplifies the insertion of zero-thickness CIEs between adjacent triangular solid elements. This study assumes that NAs will not break, hence no CIEs are inserted within NA. The inserted CIEs are respectively RBCA CIEs, new mortar CIEs, old mortar, RBCA–old mortar CIEs (old ITZ CIEs), new mortar–old mortar CIEs (new ITZ CIEs), and NCA–new mortar CIEs (new ITZ CIEs). With the RR of recycled concrete set to 50%, a schematic diagram of the inserted CIEs for each phase is shown in Figure 3, with a total of 23,008 zero-thickness CIEs inserted.

### 2.3. Model Constitutive

**Figure 4:**
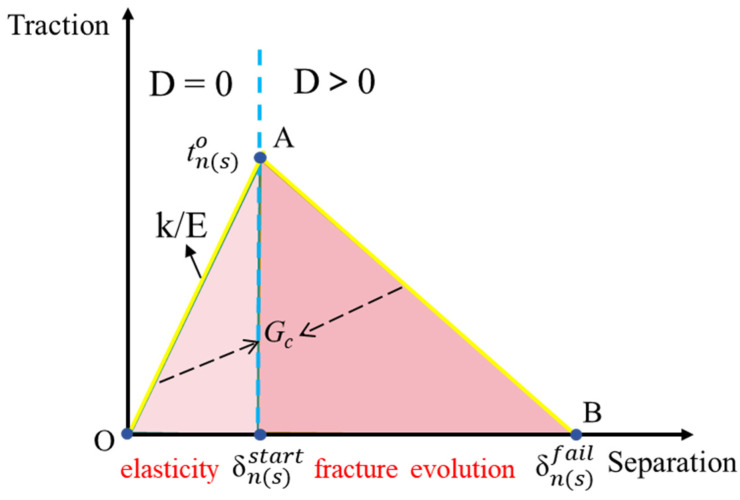
Constitutive relations of traction and separation.

**Figure 5:**
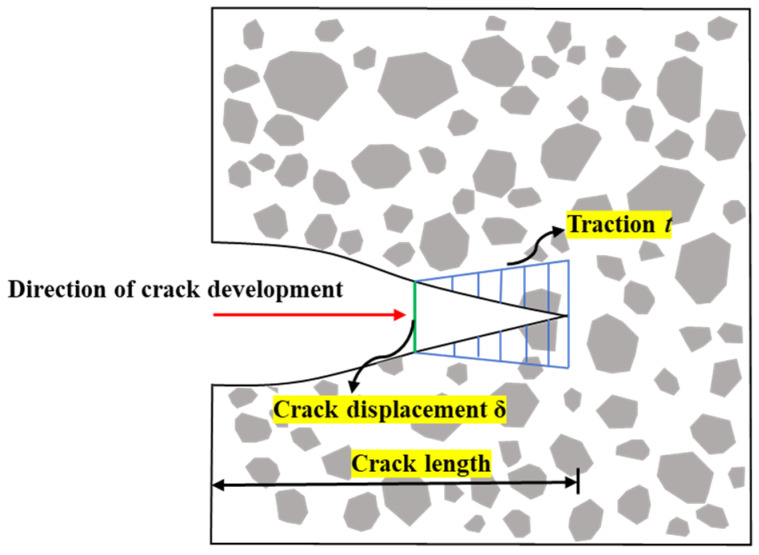
The development process of cracks in the opening mode deformation pattern [37].

The CZM is based on the traction–separation criterion. It simplifies the complex fracture process by expressing the “relative displacement–force” relationship between two surfaces, which reflects the interactions between molecules and atoms at the macroscopic level. The deformation mode of two-dimensional CIEs is mainly characterized by opening. This study uses a bilinear constitutive curve, as shown in Figure 4. $\delta_{n}^{start}$ and $\delta_{s}^{start}$ respectively represent the initial cracking displacements in the normal and tangential directions, while $\delta_{n}^{fail}$ and $\delta_{s}^{fail}$ at point B respectively represent the final fracture displacements in the normal and tangential directions. The whole process is divided into the elastic phase (OA segment) and fracture development phase (AB segment). In the OA phase, internal cracks in the specimen do not propagate, and the slope at this time represents the material’s initial tensile stiffness *K_n_* and initial shear stiffness *K_s_*. The AB phase is the crack fracture process zone (see Figure 5). To control the deformation of CIEs during the elastic phase, this section determines the material’s stiffness *K* through a trial-and-error method [36].

Because the inserted CIEs have zero thickness, it is necessary to assume an initial thickness in ABAQUS software, setting the initial thickness of CIEs to 1 mm. As the crack displacement cannot be predetermined, the fracture energy is used to represent the crack displacement. Benzeggagh et al. [38] used calibration experiments to determine the fracture energies for type I, type II, and mixed-type fractures. The total fracture energy expression for the material is as follows in Equation (4). The expression for crack displacement and material fracture energy is as follows in Equation (5). It can be seen from Figure 4 that the fracture energy (*G_c_*) of the material is equivalent to the area of the triangle.

(4) $$G_{c}=G_{nc}+(G_{tc}-G_{nc}){\left(\frac{G_{tc}}{G_{nc}}\right)}^{m}$$

(5) $$\delta^{f}=\frac{2G_{c}}{T}$$

In the equation, $G_{nc}$ represents the fracture energy associated with an opening deformation mode; $G_{tc}$ represents the fracture energy associated with a shearing deformation mode; $G_{c}$ is the total fracture energy, with m optimal that is set to 2.6; and T is the tensile strength against cracking. For the two-dimensional CZM, only the material’s normal and tangential damage evolution is considered, and the relationship between stress and strain is expressed by Equation (6) or (7):

(6) $$t=\left\{\begin{array}{c} t_{n}\\ t_{s} \end{array}\right\}=\left[\begin{array}{cc} k_{nn} & k_{ns}\\ k_{ns} & k_{ss} \end{array}\right]\left\{\begin{array}{c} \delta_{n}\\ \delta_{s} \end{array}\right\}=K\delta$$

(7) $$t=\left\{\begin{array}{c} t_{n}\\ t_{s} \end{array}\right\}=\left[\begin{array}{cc} E_{nn} & E_{ns}\\ E_{ns} & E_{ss} \end{array}\right]\left\{\begin{array}{c} \varepsilon_{n}\\ \varepsilon_{s} \end{array}\right\}=E\varepsilon$$

where *K* is the stiffness matrix, δ represents displacement, E is the modulus of elasticity matrix, and ε is strain. ε = $\frac{\delta }{h}$, where *h* is the constitutive thickness of the material, taken as 1 mm.

### 2.4. Fracture Criterion

Upon reaching the damage initiation criterion, the material will fail according to the defined damage evolution law, corresponding to point A in Figure 4. In this section, the quads damage criterion is selected as the crack fracture standard. When 1 ≤ *f* ≤ 1.05, the crack fractures are expressed as in Equation (8):

(8) $$1\;\leq \;{\left\{\frac{\langle t_{n}\rangle }{t_{n}^{o}}\right\}}^{2}+{\left\{\frac{t_{s}}{t_{s}^{o}}\right\}}^{2}\leq 1.05$$

where $t_{n}^{o}$ and $t_{s}^{o}$ represent the maximum nominal stresses of the material under pure tensile and pure shear stress states, respectively. Here, < > denotes the Macaulay bracket.

(9) $$\langle t_{n}\rangle =\left\{\begin{array}{c} t_{n}\;t_{n}\geq 0\;(for\;tension)\\ 0\;t_{n}<0\;(for\;compression) \end{array}\right.$$

After damage occurs, the CIEs enter the softening phase. The damage parameter *D* is used to indicate the extent of damage in the CIEs, and an equivalent displacement is introduced, $\delta_{m}$. The calculation Equation is as follows:

(10) $$\delta_{m}=\sqrt{{\langle \delta_{n}\rangle }^{2}+{\delta_{s}}^{2}}$$

(11) $$D=\frac{\delta_{m}^{f}(\delta_{m}^{max}-\delta_{m}^{o})}{\delta_{m}^{max}(\delta_{m}^{f}-\delta_{m}^{o})}$$

In the Equation, $\delta_{m}^{max}$ represents the maximum equivalent displacement during the loading process, $\delta_{m}^{o}$ represents the displacement when the stress of the CIEs reaches the tensile strength, and $\delta_{m}^{f}$ represents the equivalent displacement when the material fractures, corresponding to the point in Figure 4. The value of *D* ranges from 0 to 1. When *D* = 0, the CIEs are undamaged and when *D* = 1, the CIEs are completely damaged. Based on the stiffness criterion, the calculation equations for the loading and unloading stiffness coefficients of the CIEs are as follows:

(12) $$\left\{\begin{array}{c} k_{n}=(1-D)k_{no}\\ k_{s}=(1-D)k_{so} \end{array}\right.$$

The relationship between the traction force and the damage parameter can be represented by the following expression:

(13) $$t_{n}=\left\{\begin{array}{l} (1-D)\bar{t_{n}},\;\bar{t_{n}}\geq 0\\ \bar{t_{n}},\;\bar{t_{n}}<0 \end{array}\right.$$

(14) $$t_{s}=(1-D)\bar{t_{s}}$$

where $\bar{t_{n}}$ and $\bar{t_{s}}$ represent the traction of CIEs under undamaged conditions.

## 3. Simulation Parameters

**Figure 6:**
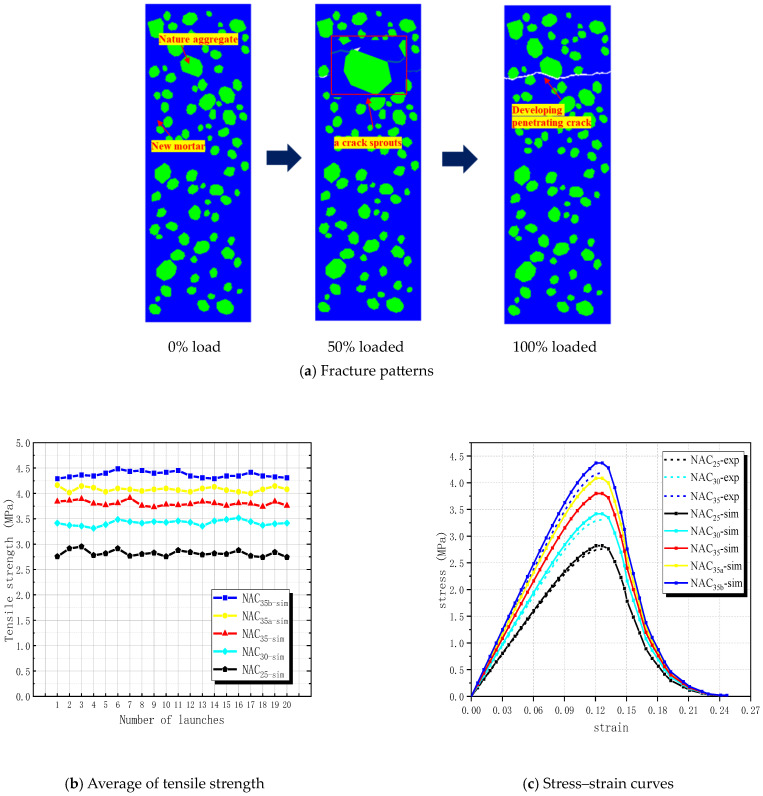
The comparison between simulation and experimental of Xu.

Due to the similar loading conditions between Xu’s tensile test [39] and this study, we conducted a mesoscale numerical simulation of Xu’s experiment. This allowed us to determine the fracture parameters of the constituent materials within the concrete, providing a data reference for simulating RBCA concrete. The simulation results are compared with the experimental results, providing a reference for simulating RBCA concrete. The mix proportions used in the experiment are presented in Table 2. The coarse aggregate comprises continuous graded gravel with a maximum aggregate diameter of 20 mm, while the sand is natural river sand, with medium granularity and particle grading falling within Zone II. Eight bolts, uniformly distributed, were used in the experiment to connect the load transfer steel plate with the bonded steel plate. The incorporation of fly ash in the preparation of C20 grade concrete had a certain impact on the experimental results. Therefore, this control experiment only selected concrete of grades C25, C30, and C35 for comparison. Three sets of ordinary concrete models with dimensions of 100 mm × 300 mm were established, yielding measured tensile strengths of 2.76 MPa, 3.31 MPa, and 4.19 MPa, respectively. The most typical fracture pattern observed is illustrated in Figure 6.

The established NAC model, depicted in Figure 6 and with a coarse aggregate volume fraction of 40%, was calibrated by referring to some material parameters from Yu [21] and using the trial-and-error method. The resulting material parameters are listed in Table 3. Employing the traction–displacement criterion, a bilinear constitutive model (refer to Figure 4) was adopted, assigning material properties to aggregate, mortar, mortar–mortar cohesive interface elements (CIEs), and aggregate–mortar CIEs. The fracture process of the model under loading is illustrated in Figure 6a. At 50% loading, tiny cracks emerge in the transition zone between the upper aggregate–mortar interface of the model. Upon reaching 100% loading, numerous tiny cracks connect with each other, forming penetrating cracks that divide the specimen into two parts. The crack propagation zone predominantly involves mortar–mortar CIEs and aggregate–mortar CIEs. The final position and shape of the cracks are largely consistent with the experiment.

In this section, five parameter sets are established based on the experiment, with 20 models drawn for each set. The average values of the results were taken, and the results obtained from the experiment and simulation are shown in Figure 6b,c. NAC_25-exp_ and NAC_25-sim_ represent the experimental and simulated results of the C25 grade concrete, respectively. Due to the limited stiffness of the experimental equipment, the stress–strain curve measured only shows the ascending stage, which does not affect the final experimental results. The tensile strengths measured for NAC_25-sim_, NAC_30-sim_, NAC_35-sim_, NAC_35a-sim_, and NAC_35b-sim_ are 2.82 MPa, 3.42 MPa, 4.37 MPa, 4.08 MPa, and 3.8 MPa, respectively. The errors, compared with the experimental values, are 2.12%, 3.22%, 4.30%, 2.63%, and 9.31%, respectively. The results, after discarding the maximum error, indicate that the established model demonstrates a higher level of reliability.

**Figure 7:**
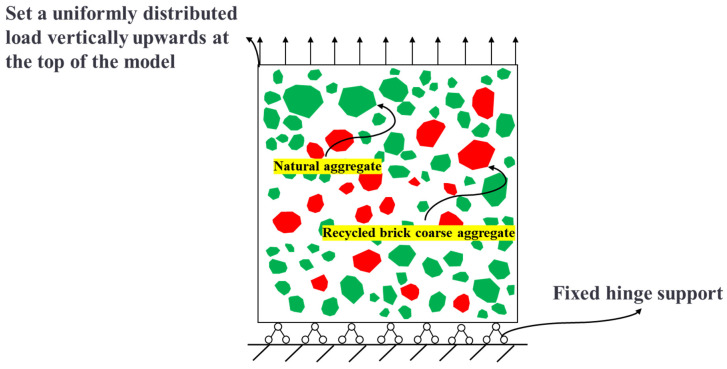
Boundary conditions of RBCA concrete.

To improve the computational efficiency of the model, the model dimensions are standardized to a uniform size, that is, the cross-sectional dimensions of the NAC and RBCA concrete models are 100 mm × 100 mm. Based on the findings of previous scholars, the elastic modulus of the old interface transition zone is approximately 0.7 to 0.8 times that of the old mortar, while the elastic modulus of the new interface transition zone is about 0.8 to 0.9 times that of the new mortar [40]. For this study, the upper limit values from these references are adopted. Concurrently, the elastic modulus of the old mortar is set to 0.85 times that of the new mortar. The material properties of RBCA concrete are presented in Table 4. The boundary conditions of the model are shown in Figure 7.

**Figure 8:**
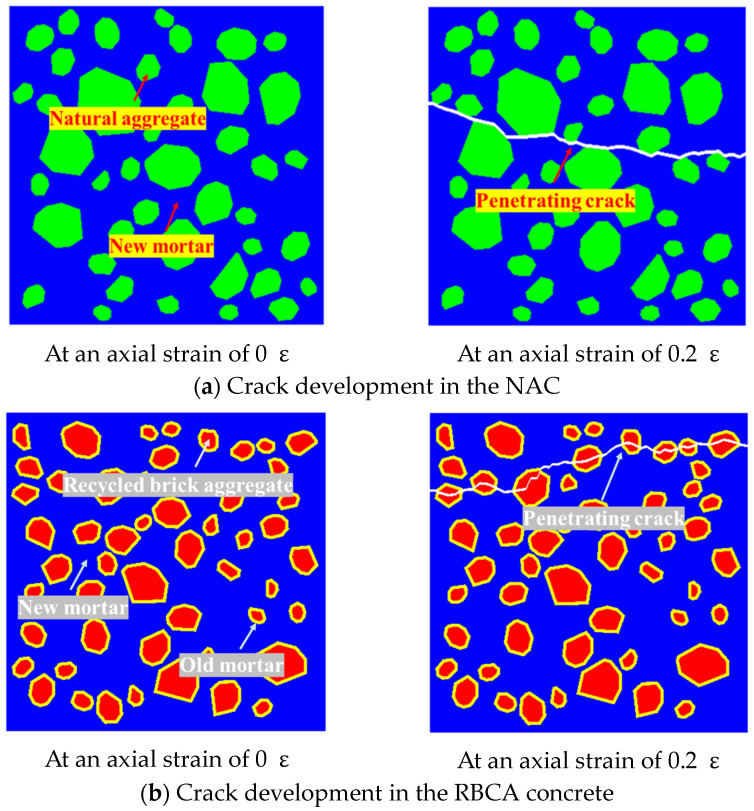
Fracture patterns of NAC and RBCA concrete.

Figure 8 illustrates the fracture diagrams of NAC and RBCA concrete with a 100% replacement rate under tensile stress. The parameters for NAC_30_ are obtained from Table 3, while those for RBCA concrete are derived from Table 4, with both having the same aggregate volume fraction. A distinct disparity in fracture patterns between NAC and RBCA concrete is evident. In NAC, cracks predominantly propagate through new mortar cohesive interface elements (CIEs) and new interface transition zone (ITZ) CIEs, resulting in through-cracks and with their respective crack paths accounting for approximately 42.3% and 57.2%, respectively. Conversely, cracks in RBCA concrete extend through new mortar CIEs, old mortar CIEs, new–old mortar ITZ CIEs, recycled brick coarse aggregate (RBCA)–old mortar ITZ CIEs, and brick aggregate CIEs. There is a notable preference for propagating through RBCA–old mortar ITZ CIEs and brick aggregate CIEs, where the crack path reaches up to about 72.5%. Wang [41] also studied the tensile properties of RBCA concrete, observing that, during the splitting of recycled concrete specimens, the fracture primarily involves the damage of the recycled brick aggregate itself, along with the destruction of cement mortar and the interface transition zone. This observation aligns with the simulation results.

**Figure 9:**
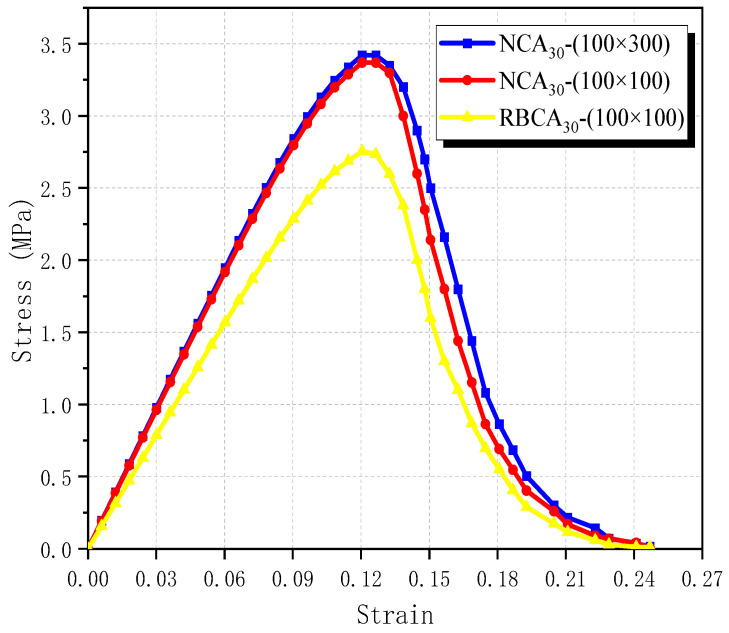
Simulated stress–strain curves of NAC and RBCA concrete.

In Figure 9, the simulated stress–strain curve shows that the strength of the NAC model decreases to 3.36 MPa, a reduction of approximately 1.75%, and that the modulus of elasticity decreases from 28.5 MPa to 28 MPa when the cross-sectional size is altered from 100 mm×300 mm to 100 mm×100 mm. It is observed that specimens with smaller cross-sectional sizes experience a faster decline in the softening phase, attributed to the fact that the smaller the loading of the specimen, the more susceptible it is to being pulled apart. After replacing NA with RBCA, the tensile strength of RBCA concrete drops to 2.75 MPa, with a modulus of elasticity of 22.92 MPa (a reduction of about 18.14%). Compared with the NAC_30_ with a size of 100 mm × 100 mm, its tensile strength decreases by about 18.48%. Hence, it is apparent that the incorporation of brick aggregate markedly diminishes the tensile strength of recycled concrete. This result is consistent with the findings of numerous scholars [14,42,43], further validating the accuracy of the RBCA concrete model. The article then analyzes the main factors affecting the tensile performance of RBCA concrete from multiple perspectives.

## 4. Analysis and Discussion

### 4.1. Impact of Brick Aggregate RR and Aggregate Distribution on the Tensile Fracture of RBCA Concrete

**Figure 10:**
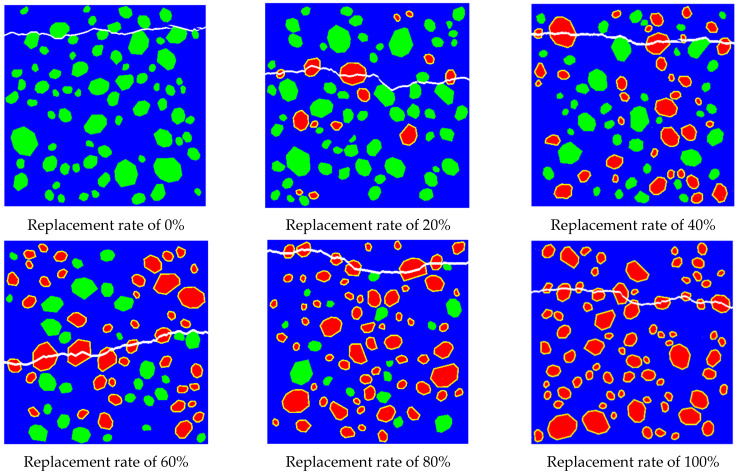
Fracture patterns of RBCA concrete at different replacement rates.

In this section, brick aggregate replacement rates (RRs) are set to 0%, 20%, 40%, 60%, 80%, and 100%, with the cross-sectional dimensions of the specimens set at 150 mm × 150 mm. Utilizing the material parameters from Table 4, researchers conducted simulations of tensile cracks in RBCA concrete. Figure 10 illustrates the typical fracture patterns of RBCA concrete under tensile stress. Initially, micro-cracks emerge around the recycled brick coarse aggregate (RBCA) at the interface transition zone (ITZ). With the gradual increase in load, these cracks propagate from the ITZ and mortar into the interior of the brick aggregate, ultimately leading to the specimen splitting in two, forming transverse through-cracks. Compared with natural aggregate concrete (NAC), RBCA concrete containing brick aggregate exhibits a higher propensity to form transverse cracks, primarily due to the brittleness of the brick aggregate, which allows cracks to penetrate its interior.

**Figure 11:**
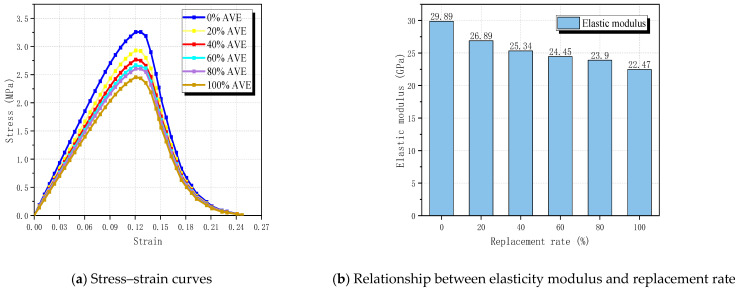
Effect of brick aggregate replacement rate on the tensile properties of RBCA concrete.

Figure 10 demonstrates significant differences in crack locations within RBCA concrete at different RRs, with cracks predominantly expanding in regions concentrated with brick aggregate. Compared with natural aggregates, RBCA has a lower initial strength, and cracks are more likely to propagate through the RBCA when subjected to loads. Therefore, it can be inferred that the crack propagation location is closely related to the placement of the brick aggregate. To investigate whether the placement position influences the tensile performance of RBCA concrete, specimens with the same replacement rate are grouped together. Each group randomly places 20 RBCA concrete models with the same RR, assigns the same material properties, and conducts simulations of tensile experiments. The results are then averaged to obtain the tensile outcomes of RBCA concrete at different RRs, as depicted in Figure 11.

In Figure 11a, the stress–strain curves of specimens at different replacement rates (RRs) are depicted. It is evident that the stress–strain curves of natural aggregate concrete (NAC) and RBCA concrete are largely consistent, indicating that phase distribution has minimal impact on RBCA concrete performance. However, the placement of coarse aggregate exerts a minor influence on the elastic modulus of RBCA concrete while significantly affecting the shape of cracks.

At an RR of 20%, the tensile strength of RBCA concrete is 2.93 MPa, marking a reduction of approximately 9.85% compared with NAC, with the elastic modulus decreasing from 29.88 GPa to 26.89 GPa. This decline can be attributed to the inclusion of broken bricks, which diminish the bearing capacity of RBCA concrete. The low strength and high brittleness of the brick aggregate result in weakened tensile performance. As the RBCA replacement rate escalates from 20% to 60%, the tensile strength of RBCA concrete decreases from 2.93 MPa to 2.61 MPa, and the elastic modulus diminishes to 24.45 GPa, representing nearly an 18.17% reduction compared with NAC. Upon reaching an RBCA replacement rate of 100%, the strength of RBCA concrete drops to 2.46 MPa, marking a reduction of approximately 24.31% compared with NAC, with the elastic modulus declining to 22.47 GPa.

From the simulation results, it is statistically inferred that the average number of cracks sequentially passing through brick aggregate is 0, 4.2, 5.45, 6.5, 7.3, and 10.55. Therefore, it is evident that, with the increase in brick aggregate replacement rate, the tensile strength and elastic modulus of RBCA concrete gradually decrease, while the proportion of crack length passing through the brick aggregate to the total crack length increases. This conclusion is consistent with the experimental findings of other scholars [44,45,46].

### 4.2. Impact of the Mortar Strength on the Tensile Fracture of RBCA Concrete

The mechanical properties of recycled aggregate concrete are intricately linked to the strength of the mortar [47]. Hence, to investigate the impact of mortar strength on the tensile properties of RBCA concrete, this section establishes the replacement rate of recycled brick aggregate at 100%. The pore shapes are set as circular, with a porosity rate of 2% and pore size of 3 mm. Based on Table 4, the initial values are $E_{new(old)\;mortar}$ = 29,000 (24,650) and $k_{new(old)\;motar-new(old)\;mortar\;CIEs}$ = 320,000 (272,000). Set $E_{new(old)\;mortar}^{\prime }$ = ${\mu E}_{new(old)\;mortar}$ and $k_{new(old)\;motar-new(old)\;mortar\;CIEs}^{\prime }$ = ${\delta k}_{new(old)\;motar-new(old)\;mortar\;CIEs}$, where (μ,δ) = (0.55, 0.55) and increases to (1.3, 1.3) with an increment of 0.15, indicating the enhancement of new mortar and old mortar strength.

**Figure 12:**
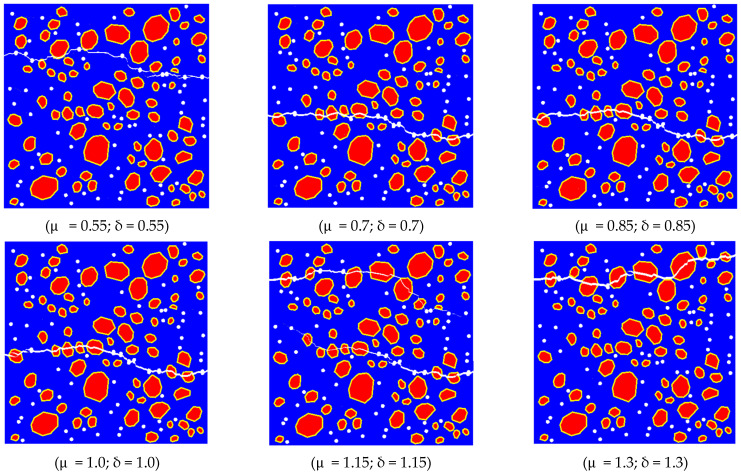
RBCA concrete fracture patterns with different mortar parameters.

In Figure 12, the fracture patterns of RBCA concrete under different mortar parameters are depicted, revealing the significant influence of mortar strength on crack propagation direction in RBCA concrete. For (μ, δ) = (0.55, 0.55), cracks propagate solely through the pores, new mortar, and old mortar, indicating that the mortar strength is lower than that of the brick aggregate. Conversely, for (μ, δ) = (0.7, 0.7) to (μ, δ) = (1.0, 1.0), the crack patterns remain relatively consistent, suggesting a minor effect of mortar strength within this range on the crack shape in RBCA concrete. However, when (μ, δ) ≥ (1.15, 1.15), a notable variation in RBCA concrete crack shape is observed, with a decrease in the proportion of mortar in the cracks and an increase in the proportion of brick aggregate.

**Figure 13:**
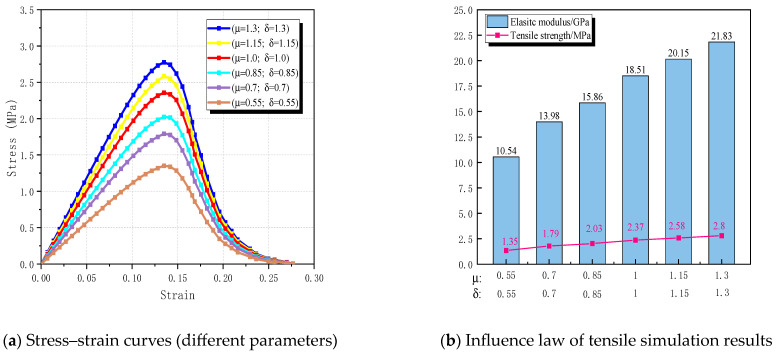
Effect of mortar strength on tensile properties of RBCA concrete.

In Figure 13, the stress–strain curve shapes under different mortar strengths are generally consistent. As (μ, δ) increases from (0.55, 0.55) to (1.3, 1.3), the tensile strength of RBCA concrete rises from 1.35 MPa to 2.8 MPa, with respective increments of 24.58%, 11.82%, 14.35%, 8.14%, and 7.86% between adjacent parameters. Concurrently, the corresponding elastic modulus increases from 10.54 GPa to 21.83 GPa, marking a 51.72% increase. This indicates that, as mortar strength increases, the growth rates of RBCA concrete’s tensile strength and elastic modulus exhibit a diminishing trend. This suggests that excessive enhancement of mortar strength does not significantly improve RBCA concrete’s tensile performance, while reducing mortar strength can considerably weaken it.

### 4.3. Impact of Pore Structure on the Tensile Fracture of RBCA Concrete

**Figure 14:**
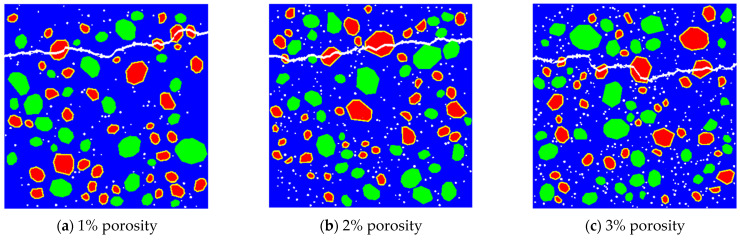
Fracture patterns for RBCA concrete with different porosities.

There are many reasons for the formation of pores in concrete, such as incomplete hydration reactions, capillary pores formed by water evaporation, and air entrapped during mixing [48]. Wang’s research [49] indicates that pore structure significantly affects the mechanical properties of concrete. Therefore, this section aims to analyze the effect of pore structure on the tensile performance of RBCA concrete, considering factors such as porosity, pore size, and pore shape. By fixing the replacement rate of RBCA at 50% and using specimens with cross-sectional dimensions of 150 mm × 150 mm, simulations under different porosities, with circular pore shapes and with pore sizes ranging from 1–2 mm were conducted. The tensile simulation of RBCA concrete specimens, based on the properties assigned to each phase material, as per Table 4, revealed typical fracture patterns as shown in Figure 14. It was observed that, at porosities ≤3%, the tensile fracture pattern of RBCA concrete consistently showed transverse cracks, with the crack length significantly increasing and the crack path becoming more tortuous as porosity increased. Analyzing the reasons, under tensile stress, compared with other material phases, pores are more prone to the formation of microcracks. Therefore, increasing the number of pores elevates the probability of crack formation. As the load increases, cracks at the pores connect with cracks in other material phases, gradually forming continuous cracks. From Figure 17a, it is evident that, under different porosities, the stress–strain curves of RBCA concrete maintain a consistent shape, with tensile strengths corresponding to 0%, 1%, 2%, and 3% porosities being 2.69 MPa, 2.39 MPa, 2.14 MPa, and 2.07 MPa, respectively. In Group A of Figure 18, the elastic modulus decreases from 25.01 GPa to 19.28 GPa, a reduction of approximately 22.91%. This indicates that an increase in porosity somewhat reduces the specimen’s tensile strength and elastic modulus.

**Figure 15:**
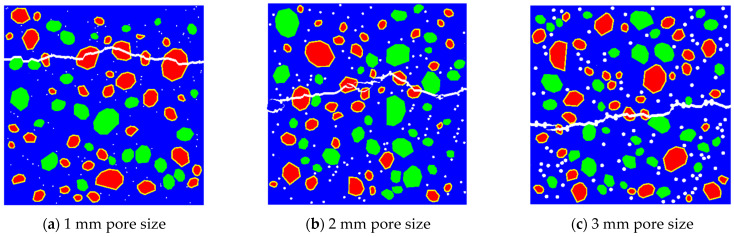
Fracture patterns for RBCA concrete with different pore sizes.

Random placement of 115 circular pores with different diameters (1 mm, 2 mm, and 3 mm) was simulated for RBCA concrete under tension, with typical fracture patterns shown in Figure 15. When the pore size increased to 2 mm, there was a noticeable change in the direction of crack propagation, appearing as discontinuous curves. This change is attributed to the random placement of pores; as the pore size increases, it becomes easier for adjacent pores to connect and form continuous cracks. With increasing load, the cracks formed by the other phases intersect with those between the pores, creating long continuous cracks, while cracks that do not intersect remain independent. When the pore size increased to 3 mm, the intersections also exhibited discontinuities. The random placement of pores makes the number of non-intersecting cracks uncertain. According to simulation results, with a constant number of pores and when the pore size is ≥2 mm, the effect of pore size on the number of non-intersecting cracks is minor, and the placement of pores and aggregates has a greater impact. With an increase in pore size to 3 mm, the tensile strength of RBCA concrete decreased from 2.69 MPa to 2.18 MPa, and the elastic modulus dropped to 20.31 GPa, a reduction of about 18.79%.

**Figure 16:**
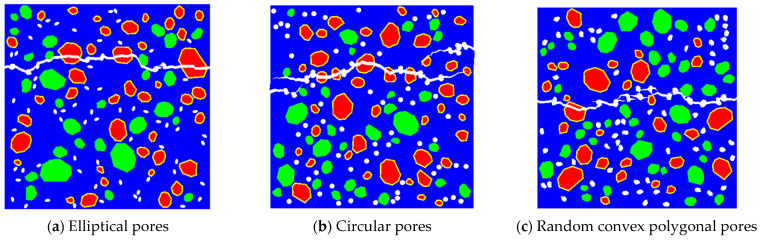
Fracture patterns for RBCA concrete with different pore shapes.

**Figure 17:**
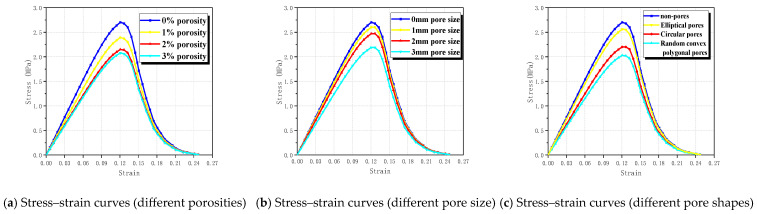
Stress–strain curves of RBCA concrete under different influencing factors.

**Figure 18:**
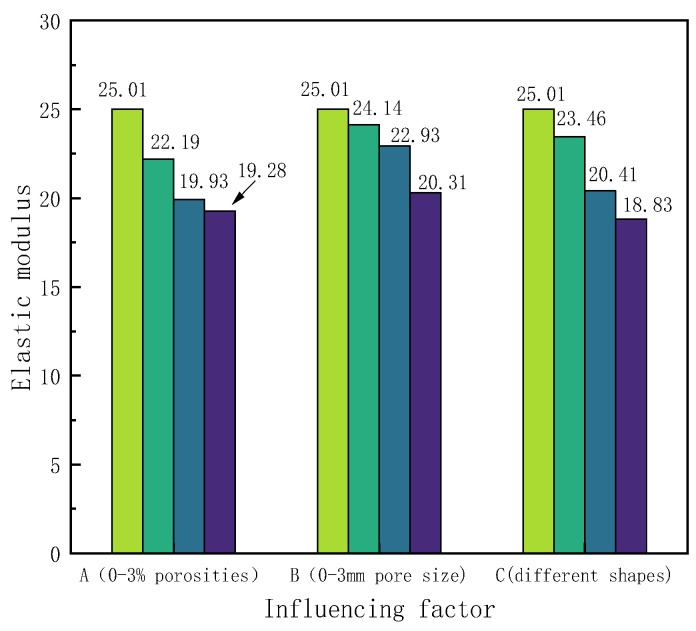
Influence of different factors on the tensile properties of RBCA concrete.

To study the impact of pore shape on RBCA concrete’s tensile properties, 72 pores with a maximum aperture of 4 mm, in shapes of circles, convex polygons, and ellipses, are randomly distributed, revealing distinct crack patterns as shown in Figure 16. Cracks containing circular pores showed two discontinuities, consistent with the previous findings. Cracks with convex polygonal pores exhibit numerous discontinuous fractures at areas of pore concentration, and the closer the lateral pores are to each other, the more likely cracks are to form. The elliptical pores, with a minor to major axis ratio of 0.6, showed better continuity between cracks without significant splitting. Figure 17c demonstrates that, under similar conditions, the pore shape significantly affects RBCA concrete’s tensile strength; compared with circular and elliptical pores, convex polygonal pores had the greatest impact on RBCA concrete’s tensile properties, with tensile strength dropping from 2.69 MPa to 2.02 MPa and the elastic modulus to 18.83 GPa (See Figure 18), a 24.71% reduction.

**Figure 19:**
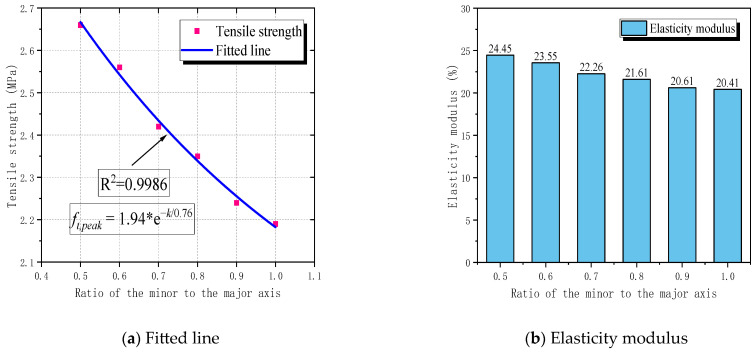
Effect of different ratios of the minor to the major axis of elliptical pore on the tensile properties of RBCA concrete.

This study found that the ratio of the minor to the major axis of elliptical pores affects the tensile properties of RBCA concrete. Therefore, tensile simulations were carried out on pore models with different ratios of the minor to the major axis, setting the long axis size to 4 mm, with different ratios of the minor to the major axis (K) ranging from 0.5 to 1.0, with an increment of 0.1. The final nonlinear fitting result is shown in Figure 19a, with a correlation of 0.9986. The fitting curve is an expression related to EXPDEC1, as seen in Equation (15).

(15) $$f_{t}\;=\;1.94\;\times \;e^{-\frac{k}{0.76}}\;R^{2}=0.9986$$

Here, K represents the ratio of the minor to the major axis of elliptical pores, and *f_t_* represents the specimen’s tensile strength. It is evident that, when the ratio of the minor to the major axis is greater than or equal to 0.5, and as the K value of elliptical pores increases, the tensile strength of RBCA concrete gradually decreases. The tensile strengths corresponding to each K value are 2.66 MPa, 2.56 MPa, 2.42 MPa, 2.35 MPa, 2.24 MPa, and 2.19 MPa, respectively, with the modulus of elasticity decreasing from 24.45 GPa to 20.41 GPa. When analyzing the reason for this, with the long axis size fixed, we found that increasing K subsequently increases the short axis size from 2 mm to 4 mm, causing a gradual decrease in the mechanical properties of RBCA concrete. This conforms to the conclusion that the size of the pores is inversely proportional to the tensile strength and modulus of elasticity of RBCA concrete. As the shape of the pores gradually changes from elliptical to circular, the tensile strength and modulus of elasticity values of models with elliptical pores gradually approach those of models with circular pores. Therefore, it can be concluded that, when the ratio of the minor to the major axis of elliptical pores is 0.5 ≤ K < 1, the impact of pore shape on the tensile properties of RBCA concrete is as follows: random convex polygonal pores > circular pores > elliptical pores. In summary, for concrete specimens containing RBCA under tensile stress, the crack shapes in the model are divided into continuous cracks and split cracks, which are not only related to the placement of aggregates and pores but also to the porosity, pore size, and shape of the pores. Porosity and pore size are negatively correlated with the tensile strength and modulus of elasticity of RBCA concrete, and under the same conditions, elliptical pores have the least impact on the tensile properties of RBCA concrete.

### 4.4. Impact of the ITZ Strength between Brick Aggregates and Mortar on the Tensile Fracture of Concrete

Several studies [40,50,51,52] have indicated that the ITZ strength between aggregate and mortar is a key factor in controlling the macroscopic mechanical properties of concrete. Xiao [53] tested the properties of the ITZ in recycled aggregate concrete using nanoindentation, and their results show that there are pores and a high concentration of calcium hydroxide at the ITZ in RAC. The thicknesses of the new and old ITZ were found to be 40–50 μm and 55–65 μm, respectively. Liu [54], in studying the tensile bond strength of concrete with recycled aggregate replacing natural aggregate, found that the tensile bond strength of RA concrete increased by 5–49% compared with NA. However, there is relatively little research on the ITZ between recycled brick aggregate and mortar. To independently analyze the influence of ITZ strength at the recycled RBCA site on the tensile properties of concrete, this section excludes natural aggregate. Accordingly, the replacement rate of RBCA concrete is set to 100%, maintaining the same pore structure as discussed in Section 4.2. The stiffness, k, is used as a variable, based on the initial values of $k_{New\;mortar-old\;mortar\;CIEs}$ = 288,000 and $k_{RBCA-old\;mortar\;CIEs}$ = 217,600 in Table 4. Assuming $k_{New\;mortar-old\;mortar\;CIEs}^{\prime }$ = ${\eta k}_{New\;mortar-old\;mortar\;CIEs}$ and $k_{RBCA-old\;mortar\;CIEs}^{\prime }$ = ${\gamma k}_{RBCA-old\;mortar\;CIEs}$, the range of (η,γ) changes from (0.1, 0.15) to (0.3, 0.35), (0.5, 0.55), (0.7, 0.75), (0.9, 0.95), and (1.1, 1.15) to indicate the enhancement of strength at the ITZ.

**Figure 20:**
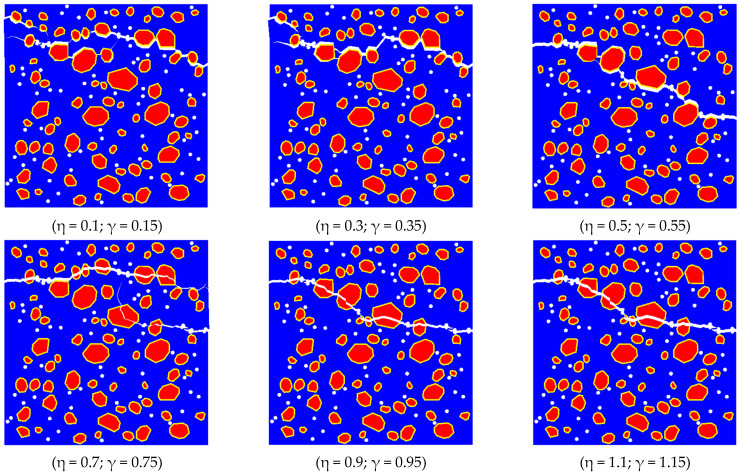
RBCA concrete fracture patterns with different ITZ–CIE parameters.

The observations from Figure 20 reveal notable shifts in the crack propagation direction under varying ITZ stiffness conditions. When the stiffness and maximum traction force at the ITZ were lower than those of RBCA, specifically η ≤ 0.5 and γ ≤ 0.55, cracks predominantly initiated at the ITZ rather than within the aggregates, due to the lower stiffness, elastic modulus, and maximum traction force at the ITZ compared with RBCA. As the stiffness increased to η ≥ 0.7 and γ ≥ 0.75, cracks began propagating inside the RBCA. The crack patterns at (0.9, 0.95) and (1.1, 1.15) were relatively similar due to minimal changes in tensile strength and elastic modulus, resulting in little alteration in the crack propagation direction under tensile stress in RBCA concrete.

**Figure 21:**
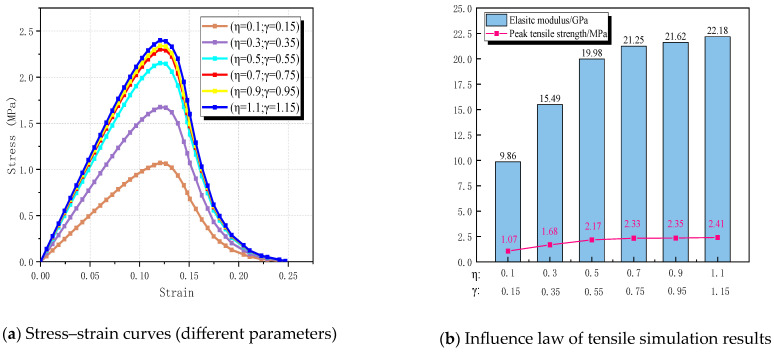
Effect of ITZ strength on tensile properties of RBCA concrete.

Figure 21a presents the predicted stress–strain curves at different ITZ strengths, clearly illustrating the significant impact of ITZ strength on the tensile properties of RBCA concrete. As the ITZ strength reduced from (1.1, 1.15) to (0.5, 0.55), the tensile strength decreased from 2.41 MPa to 2.17 MPa, a drop of about 9.96%, with a corresponding decrease in elastic modulus of about 9.92%. Further reduction in strength at the ITZ from (0.5, 0.55) to (0.1, 0.15) led to a decrease in tensile strength from 2.17 MPa to 1.07 MPa, a reduction of about 50.69%. Thus, excessively reducing the ITZ strength can significantly weaken the tensile properties of RBCA concrete, while overly enhancing the ITZ strength only slightly improves RBCA concrete’s tensile performance. Therefore, adopting appropriate reinforcement measures in engineering, such as the silicate solution soaking method employed by Qiu [55] to strengthen the ITZ of brick aggregate concrete, can optimize the performance of RBCA concrete.

### 4.5. Impact of the Brick Coarse Aggregate Volume Fraction on the Tensile Fracture of Concrete

**Figure 22:**
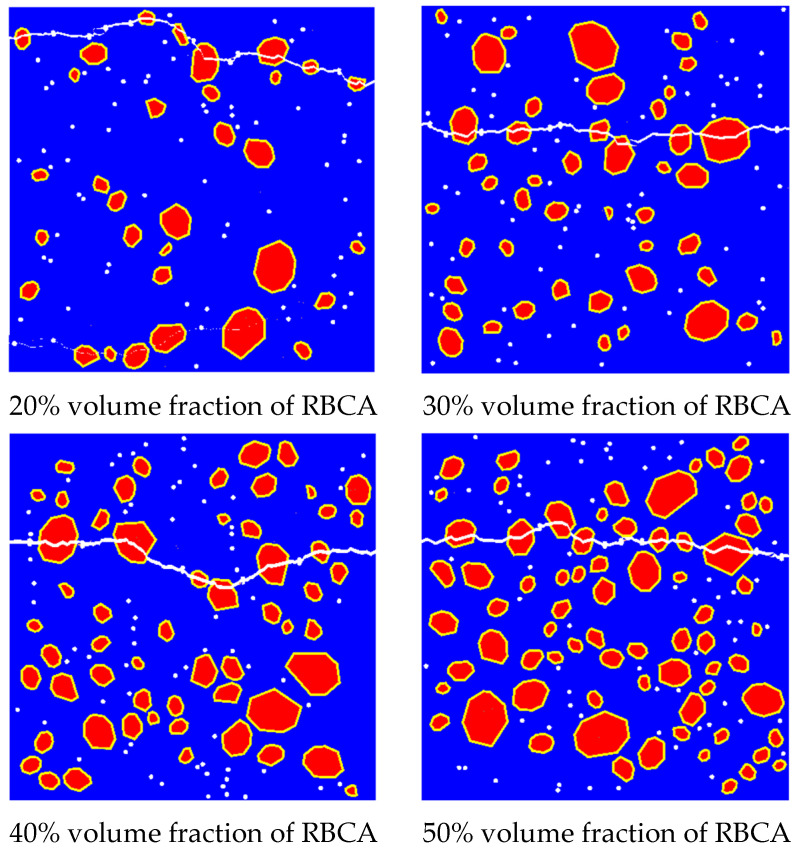
Fracture patterns for different brick aggregate volume fractions.

Uddin [56] indirectly studied the effect of aggregate volume fraction on the mechanical properties of concrete by controlling the aggregate particle size. Their results indicate that, as the particle size of brick aggregate increases, the splitting tensile strength of RC gradually decreases. However, there is currently no experimental data available to study the impact of different RBCA volume fractions on the tensile strength of concrete. In this section, the volume fractions of brick aggregate were set at 20%, 30%, 40%, and 50% in order to investigate their impact on the mechanical properties of RBCA concrete. To eliminate the influence of natural coarse aggregate (NCA) on the tensile performance of RBCA concrete, the brick aggregate replacement rate was set to 100%, with a pore size of 1 mm, porosity of 1%, and circular pore shape. Figure 22 illustrates the fracture patterns of the models, revealing that models with a 20% volume fraction exhibit relatively smaller crack widths when compared with those with volume fractions of 30% to 50%. The average number of cracks passing through the brick aggregate for volume fractions of 20% to 50% were, respectively, 5.65, 6.2, 6.45, and 8.25. It is evident that, when the volume fraction of the brick aggregate is between 20% and 40%, the number of cracks traversing the brick aggregate tends to stabilize, with an average value of 6.1.

**Figure 23:**
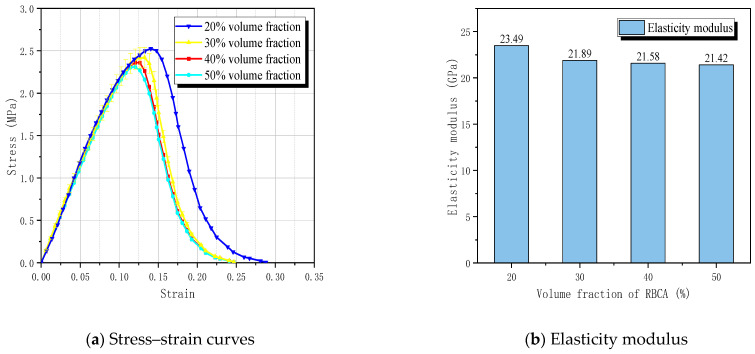
Predicted results for different aggregate volume fractions.

The simulated results, depicted in Figure 23, indicate that the average tensile strengths of RBCA concrete with different volume fractions (20%, 30%, 40%, 50%) were 2.52 MPa, 2.43 MPa, 2.38 MPa, and 2.30 MPa, with reductions ranging from approximately 8.73% to 3.36%. Correspondingly, the modulus of elasticity values were 23.49 GPa, 21.89 GPa, 21.58 GPa, and 21.42 GPa, with reductions ranging from approximately 8.81% to 0.74%.

From the simulation results, it is evident that the volume fraction of RBCA has a minimal effect on the elastic modulus of concrete. This is because the strength of the mortar exceeds that of the brick aggregates, with the concrete primarily in an elastic state before cracks appear, where the mortar bears the load. Consequently, increasing the volume fraction of brick aggregates has a limited impact on the specimen’s elastic modulus. However, the decrease in tensile strength of RBCA concrete is more pronounced than that in its elastic modulus. Upon crack formation, the load borne by brick aggregates intensifies. This is compounded by the inherent low strength of brick aggregates and the weakened bonding between aggregates and new mortar due to a layer of old mortar, significantly reducing RBCA concrete’s overall bearing capacity. Therefore, it is crucial to reasonably control the volume fraction of RBCA concrete in order to optimize the mechanical properties of RC.

## 5. Conclusions

This paper investigates the tensile performance of RBCA concrete by establishing a two-dimensional cohesive zone model. The study analyzes the effects of RBCA replacement rate, porosity, pore size, pore shape, interfacial transition zone (ITZ) strength, mortar strength, and brick aggregate volume fraction on the tensile performance of RBCA concrete. The main conclusions drawn are as follows:

(1) As the replacement rate of RBCA increases, the tensile strength and elastic modulus of RC gradually decrease, and the proportion of the crack length passing through the brick aggregates relative to the total crack length increases. Under the same conditions, the distribution location of the different material phases has a minimal impact on the elastic modulus of the recycled brick aggregates but significantly affects the direction of crack propagation.
(2) The tensile properties of RBCA concrete are greatly influenced by low-strength mortar; however, excessively increasing the mortar’s strength does not significantly enhance the tensile properties of RBCA concrete. The effect of the pore structure on the tensile properties of RBCA concrete should not be ignored during the simulation. Increasing porosity and pore size will reduce both the elastic modulus and tensile strength of RBCA concrete.
(3) Increasing the pore size at the same porosity level results in a splitting crack shape. When the pore size is ≥2 mm, the number of non-intersecting cracks is only related to the location of the pores and aggregates. Compared with circular and elliptical pores (with a minor to the major axis of 0.6), convex polygonal pores have the most significant impact on RC. When the ratio of the minor to the major axis (K) of elliptical pores is [0, 1), the tensile strength of RC is inversely proportional to K.
(4) The ITZ strength between brick aggregates and mortar is a key factor in controlling the tensile properties of RC. As the ITZ strength gradually increases, the tensile strength of RC monotonically increases. However, excessively reducing the ITZ strength significantly weakens the tensile properties of RC, while excessively increasing the ITZ strength has a relatively smaller increment in the tensile strength of RC.
(5) With the increase in the volume fraction of brick aggregates, the tensile strength and elastic modulus of RBCA concrete gradually decrease. When the volume fraction of aggregates increases from 20% to 50%, the minimum reduction in elastic modulus is about 0.74%, which has a greater impact on the tensile strength of RBCA concrete than on its elastic modulus.

## Figures and Tables

**Table 1:** NA and RA particle size distribution ratio.

Average Aggregate Size	NA Distribution Ratio (%)	RA Distribution Ratio (%)
0–5	30	30
5–10	19.51	17.7
10–15	13.24	15.23
15–20	7.25	7.07

**Table 2:** Experimental mix proportions for Xu (kg).

Specimen Notation	Water	Cement	Coarse Aggregate	Sand
NAC25	0.86	1	4.68	4.25
NAC30	0.73	1	4.36	3.08
NAC35	0.63	1	3.86	2.41

**Table 3:** Material parameters of NAC.

Specimen Notation	Aggregate (E, ν)	Mortar (E, ν)	Mortar–Mortar CIEs $\left(k,t_{n}^{o},\delta_{n}^{fail}\right)$	Aggregate–Mortar CIEs $\left(k,t_{n}^{o},\delta_{n}^{fail}\right)$
NAC25	(80, 000, 0.16)	(27, 000, 0.25)	(325, 000, 3.25, 0.1)	(325, 000, 2.6, 0.08)
NAC30	(80, 000, 0.16)	(29, 000, 0.25)	(388, 000, 3.88, 0.1)	(348, 750, 2.8, 0.08)
NAC35	(80, 000, 0.16)	(31, 000, 0.25)	(450, 000, 4.50, 0.1)	(450, 000, 3.6, 0.08)
NAC35a	(80, 000, 0.16)	(30, 000, 0.25)	(430, 000, 4.30, 0.1)	(412, 500, 3.3, 0.08)
NAC35b	(80, 000, 0.16)	(29, 500, 0.25)	(410, 000, 4.10, 0.1)	(387, 500, 3.1, 0.08)

(Notes: E—Young’s modulus (MPa), ν—Poisson’s ratio, k—rigidity (MPa), $t_{n}^{o}$—maximum traction (MPa), $\delta_{n}^{fail}$—maximum separation displacement (mm)).

**Table 4:** RBCA concrete model material parameters.

Element Type	E	ν	k	$t_{n}^{o}$	$\delta_{n}^{fail}$
NCA	80,000	0.16	-	-	-
RBCA	17630	0.22	-	-	-
New mortar	29,000	0.25	-	-	-
Old mortar	24,650	0.25	-	-	-
RBCA CIEs	-	-	192,000	1.92	0.1
NA–New mortar CIEs	-	-	297,000	3.71	0.08
RBCA–old mortar CIEs	-	-	217,600	2.72	0.08
New mortar–old mortar CIEs	-	-	288,000	2.88	0.1
New mortar–New mortar CIEs	-	-	320,000	3.20	0.1
Old mortar–Old mortar CIEs	-	-	272,000	2.72	0.1

(Notes: E—Young’s modulus (MPa), ν—Poisson’s ratio, k—rigidity (MPa), $t_{n}^{o}$—maximum traction (MPa), $\delta_{n}^{fail}$—Maximum separation displacement (mm)).

## Data Availability

The original contributions presented in the study are included in the article, further inquiries can be directed to the corresponding authors.
